# Phylogeny and Physiological Diversity of Cold-adapted Anaerobic Bacteria Isolated from Rice Field Soil in Japan

**DOI:** 10.1264/jsme2.ME22109

**Published:** 2023-05-11

**Authors:** Sachi Honma, Atsuko Ueki, Akio Ichimura, Kouki Suzuki, Nobuo Kaku, Katsuji Ueki

**Affiliations:** 1 Faculty of Agriculture, Yamagata University, Tsuruoka, 997–8555, Japan

**Keywords:** blown pack spoilage, *Clostridium estertheticum*, *Clostridium gelidum*, *Clostridium tagluense*, psychrotrophic anaerobic bacteria

## Abstract

Cold-adapted or psychrotrophic fermentative anaerobic bacteria were isolated from rice field soil in a temperate area in Japan using anaerobic enrichment cultures incubated at 5°C. Most isolates were obligately anaerobic, spore-forming rods and affiliated with different lineages of the genus *Clostridium* based on 16S rRNA gene sequences. The growth temperature ranges and physiological properties of three representative clostridial isolates (C5S7, C5S11^T^, and C5S18) were examined. Strain C5S7 grew at 0°C, but not at 20°C, and was identified as *Clostridium estertheticum*, a psychrophile isolated from spoiled, vacuum-packed, chilled meat (blown pack spoilage, BPS). Strain C5S7 produced butyrate, *n*-butanol, and abundant gases (H_2_ and CO_2_) as major fermentation products from the carbohydrates utilized. Strain C5S11^T^, which was recently described as *Clostridium gelidum* sp. nov., possessed psychrotrophic properties and grew at temperatures between 0 and 25°C. Strain C5S11^T^ was saccharolytic, decomposed polysaccharides, such as inulin, pectin, and xylan, and produced acetate, butyrate, and gases. Strain C5S18 also grew at 0°C and the optimum growth temperature was 15°C. Strain C5S18 did not ferment carbohydrates and grew in a manner that was dependent on proteinaceous substrates. This strain was identified as the psychrotolerant species, *Clostridium tagluense*, originally isolated from a permafrost sample. Collectively, the present results indicate that psychrotrophic anaerobic bacteria with different physiological properties actively degrade organic matter in rice field soil, even in midwinter, in a cooperative manner using different substrates. Furthermore, different psychrotrophic species of the genus *Clostridium* with the ability to cause BPS inhabit cultivated soil in Japan.

Microbial communities consisting of organisms adapted to cold conditions (psychrotrophs or cold-adapted microbes) thrive in various cold habitats (Katayama *et al.*, 2007; Miteva *et al.*, 2009; Madigan *et al.*, 2017). A true psychrophile has been defined as a microorganism with an optimum growth temperature of 15°C or lower, a maximum growth temperature limit of less than 20°C, and a minimum growth temperature of 0°C or lower. Microorganisms that grow at 0°C or lower with optimum growth temperatures of between 20 and 40°C are generally called psychrotolerants (Morita, 1975; Spring *et al.*, 2003). Anaerobic bacteria also inhabit anoxic cold environments, and cold-adapted obligately anaerobic bacteria have been isolated from various environments. The majority of psychrotrophic obligately anaerobic bacteria belong to the genus *Clostridium* within the family *Clostridiaceae* of the phylum *Bacillota* (=*Firmicutes*) (Rainey *et al.*, 2009; Oren and Garrity, 2021) and were originally isolated from environments permanently at sub-zero temperatures, such as permafrost, glaciers, and polar regions. True psychrophilic *Clostridium* species reported to date include *Clostridium bowmanii*, *Clostridium frigoris*, *Clostridium lacusfryxellense*, *Clostridium psychrophilum* (Spring *et al.*, 2003), *Clostridium algoriphilum* (Shcherbakova *et al.*, 2005), and *Clostridium vincentii* (Mountfort *et al.*, 1997). *Clostridium schirmacherense* (Alam *et al.*, 2006) and *Clostridium tagluense* (Suetin *et al.*, 2009) are regarded as‍ ‍psychrotolerant species. Furthermore, psychrotrophic *Clostridium* species may cause blown pack spoilage (BPS) in vacuum-packed meat under chilled storage, which is‍ ‍caused by excess gas production by anaerobic bacteria accidentally grown in these packs. Psychrophilic species, including *Clostridium estertheticum* (*Clostridium estertheticum* subsp. *estertheticum* and *Clostridium estertheticum* subsp. *laramiense*) (Collins *et al.*, 1992; Spring *et al.*, 2003; Zhang *et al.*, 2019) and *Clostridium gasigenes* (Broda *et al.*, 2000), were isolated from various samples of vacuum-packed meat. The psychrotolerant species, *Clostridium algidicarnis*, was also isolated from a sample of spoiled, vacuum-packed cooked pork (Lawson *et al.*, 1994). Therefore, the distribution of anaerobic, psychrotrophic microbes in various environments is an important research subject for food microbiology (Broda *et al.*, 2009; Wambui and Stephan, 2019; Zhang *et al.*, 2019; Wambui *et al.*, 2020). The majority of psychrophilic *Clostridium* species are closely related to each other based on 16S rRNA gene sequence similarities and are called the *Clostridium estertheticum* complex (CEC) (Wambui *et al.*, 2021, 2022). Although two subspecies names, *C. estertheticum* subsp. *estertheticum* and *C. estertheticum* subsp. *laramiense*, are valid names in the List of Prokaryotic Names with Standing Nomenclature (LPSN) (as of February 2023), the removal of the subspecies classification of *C. estertheticum* has been proposed based on whole-genome sequence (WGS)-based ana­lyses of CEC species and novel isolates (Wambui *et al.*, 2021, 2022).

Rice paddy fields in Japan are flooded during the early period of plant cultivation, resulting in the development of highly anoxic and reduced conditions, which induce the proliferation of various anaerobic bacteria and archaea, including methanogens (Conrad *et al.*, 2012). We previously investigated anaerobic bacteria inhabiting rice field soil in northern Japan from various aspects (Kaku *et al.*, 2000; Satoh *et al.*, 2002; Akasaka *et al.*, 2003). The rice fields investigated in these studies are located along the shore of the Japan Sea in a temperate region at mid-latitudes. The air temperature of the area fluctuates from approximately 30°C in summer to 0°C in winter with heavy snow fall (annual average temperature, 12.5°C) (Kaku *et al.*, 2000). Therefore, the microbial community in the surface layer soil of the area is exposed to marked variations in temperature throughout the year. However, the distribution and ecology of cold-adapted anaerobic bacteria in rice field soil in the area have not yet been examined. During the course of investigations on anaerobic microbes in rice field soil, we isolated cold-adapted, fermentative anaerobic bacteria from a soil sample using anaerobic enrichment cultures incubated at 5°C. A phylogenetic ana­lysis based on 16S rRNA gene sequences and phenotypic examinations of the isolates showed that various psychrotrophic anaerobic bacteria with different lineages and physiological properties inhabited the soil. According to the phylogenetic, genomic, and phenotypic characteristics of one of the isolates (C5S11^T^), we proposed that the strain represents a novel psychrotrophic species in the genus *Clostridium*, and *Clostridium gelidum* sp. nov. was validly described with strain C5S11^T^ as the type strain (Honma *et al.*, 2022).

In the present study, the phylogenetic and physiological diversities of these psychrotrophic anaerobic isolates from rice field soil were examined to clarify the ecological functions of psychrotrophic anaerobic species and investigate the anaerobic microbial community structure acting under cold conditions in rice field soil. The results obtained revealed that phylogenetically different, psychrotrophic fermentative anaerobic bacteria with the potential to act as decomposers of various organic compounds, even in the cold season, inhabited rice field soil.

## Materials and Methods

### Sampling of rice field soil

Soil samples were collected from the plowed layer (depth of approximately 10‍ ‍cm) of a rice field in the Rice Breeding and Crop Science Research Institute of Yamagata Integrated Agricultural Research Center of Japan (38°45′N, 139°54′E, 10–12‍ ‍m above sea level) (Kaku *et al.*, 2000; Satoh *et al.*, 2002) using core samplers (diameter of 5.08‍ ‍cm). Immediately after sampling, soil cores were packed in polyethylene bags without an air space and the bags were sealed tightly for transport to the laboratory.

### Media and cultivation conditions

Peptone-yeast extract (PY) medium was used as the basal medium for the cultivation of anaerobic bacterial isolates unless otherwise stated. PY broth contained (L^–1^) 10‍ ‍g trypticase (BD BBL), 5‍ ‍g yeast extract, 0.45‍ ‍g K_2_HPO_4_, 0.45‍ ‍g KH_2_PO_4_, 0.9‍ ‍g (NH_4_)_2_SO_4_, 0.9‍ ‍g NaCl, 0.09‍ ‍g MgSO_4_·7H_2_O, 0.09‍ ‍g CaCl_2_·2H_2_O, 0.2‍ ‍g Na_2_CO_3_, 0.3‍ ‍g L-cysteine·HCl·H_2_O (a reducing agent), and 1‍ ‍mg sodium resazurin (a redox indicator). PY medium supplemented with (L^–1^) 0.25‍ ‍g each of glucose, cellobiose, maltose, and soluble starch and 15‍ ‍g agar, designated as PY4S agar medium, was used for agar slant cultures (Satoh *et al.*, 2002; Ueki *et al.*, 2021). Before autoclaving, the pH of the medium was adjusted to 7.2–7.4 with an NaOH solution. Media were inoculated under a stream of oxygen-free mixed gas (95% N_2_/5% CO_2_). Each test tube containing the medium was closed tightly with an inner butyl rubber stopper equipped with an outer screw cap throughout the cultivation.

### Enrichment culture and isolation of psychrotrophic anaerobic microorganisms

In preliminary examinations on psychrotrophic anaerobes in the soil samples obtained from the rice field, colonies on roll-tube agar (Holdeman *et al.*, 1977) using PY4S medium often spread on the agar surface as thin and large irregular forms and produced abundant gases, causing cracks or bubbles in the agar. Therefore, 1/10 PY4S medium, in which concentrations of trypticase, yeast extract, and sugars were reduced to 1/10 of those in PY4S medium, was used for enrichment cultures (1/10 PY4S broth) as well as for the isolation (1/10 PY4S agar) of psychrotrophic anaerobic bacteria with oxygen-free mixed gas in the headspace. Regarding enrichment cultures, soil samples were 1/10 serially diluted (10^–3^–10^–6^ dilutions) using 1/10 PY4S broth under O_2_-free N_2_ gas, and each diluted sample (1‍ ‍mL) was inoculated into three tubes containing 9‍ ‍mL of 1/10 PY4S broth and incubated at 5°C. The microbial growth of each culture was monitored (for approximately one month) through the measurement of OD_660_ by inserting culture tubes (diameter of 18‍ ‍mm) directly into a spectrophotometer equipped with a test tube holder.

Each enrichment culture, which showed an increase in turbidity, was 1/10 serially diluted using a dilution solution (Satoh *et al.*, 2002) anaerobically, and 0.2‍ ‍mL of each dilution was inoculated into three tubes containing 10‍ ‍mL of 1/10 PY4S agar for the anaerobic roll-tube method (Holdeman *et al.*, 1977). After the incubation of roll-tube agar at 5°C, the colonies that developed were randomly picked up to prepare agar slant cultures in the same medium. The purity of the isolates was confirmed through the repetition of the roll-tube method and the observation of colonies on agar medium and cell morphologies by microscopy. Isolates were named with serial numbers prefixed by ‘C5S’. Since strains isolated from enrichment cultures were confirmed to be stably cultivated and transferred at 10°C, strains were generally cultivated at 10°C. Slant cultures of isolates using 1/10 PY4S agar were kept at 4°C or in a freezer at –80°C for later ana­lyses.

### Phenotypic characterization

The phenotypic characterization of isolates was performed as previously described (Ueki *et al.*, 2021; Honma *et al.*, 2022). Growth temperature ranges were tested at temperatures of 0–30°C (with intervals of 5°C) by cultivating the isolates in 1/10 PY broth containing 1‍ ‍g‍ ‍L^–1^ glucose (1/10 PYG). Cultivation at 0°C was performed by preserving culture tubes in ice water, the temperature of‍ ‍which was monitored during the incubation. Growth rates in various temperatures were assessed based on the growth curves obtained by measuring OD_660_ as shown above at appropriate time intervals during the cultivation periods.

The growth of strains in the presence of various carbohydrates was examined using PY broth as a basal medium supplemented with 5 or 10‍ ‍g‍ ‍L^–1^ of each substrate. Organic acids and amino acids were used at 30‍ ‍mM (as a final concentration in the medium). PYG medium, which contained 10‍ ‍g‍ ‍L^–1^ glucose in PY broth, was used to cultivate inocula for physiological examinations. Amino acid utilization was examined in 1/10 PY broth as the basal medium. The utilization of substrates was assessed by measuring the OD_660_ of the culture and identifying fermentation products. Cultures in PY or 1/10 PY broth were used as the controls. Cultivation experiments were performed at least in duplicate and reproducibility was confirmed for inconsistent results. Fermentation products, such as volatile fatty acids (VFAs), alcohols, and gases (H_2_ and CO_2_), were analyzed by gas chromatography (GC) (G-3000, G-5000, or G-163; Hitachi) as previously described (Ueki *et al.*, 2020, 2021). Chemotaxonomic ana­lyses were performed according to the method described by Akasaka *et al.* (2003).

### Sequencing of the 16S rRNA gene and a phylogenetic ana­lysis

DNA samples obtained from the cell biomass of isolates cultivated in 1/10 PYG at 10°C were used as templates for PCR amplification. The 16S rRNA gene sequences of the isolates were PCR-amplified using the 27f/1492r primer pair using Ampli*Taq* Gold DNA polymerase (Perkin-Elmer) and sequences were elucidated as previously described (Satoh *et al.*, 2002). The sequences of the nearly complete 16S rRNA genes of the isolates were submitted to the DDBJ/EMBL/GenBank database to search for similar sequences using the BLAST program (Altschul *et al.*, 1997). The sequences of 16S rRNA genes from closely related species were edited using SeaView software (Gouy *et al.*, 2010) and multiple alignments were performed with the CLUSTAL W program (Larkin *et al.*, 2007). A phylogenetic tree was constructed from the evolutionary distance matrix by the neighbor-joining method with MEGA11 software (https://megasoftware.net/) (Saitou and Nei, 1987; Tamura *et al.*, 2021).

### Nucleotide sequence accession numbers and strain numbers of deposited isolates

The nucleotide sequences of the 16S rRNA genes of representative isolates were deposited in DDBJ/EMBL/GenBank under the accession numbers AB539899 to AB539907. The accession numbers of the three strains mainly examined in the present study are AB539900 (strain C5S7), AB539905 (strain C5S11^T^), and AB539901 (strain C5S18). The three representative strains, C5S7, C5S11^T^, and C5S18, were deposited in NBRC as NBRC 114691, NBRC 114689^T^, and NBRC 114690, respectively. Strain C5S11^T^ was also deposited in DSMZ as DSM 112608^T^. The WGS of strain C5S11^T^ was deposited in DDBJ/EMBL/GenBank under the accession number AP024849 (GCA_019977655.1).

## Results

### Isolation of cold-adapted anaerobic bacteria from rice field soil samples

Twenty strains were obtained as pure cultures from enrichment cultures. Basic phenotypic properties (cellular characteristics, aerobic growth, growth temperature ranges, and fermentation end products) were examined for all strains. All strains grew under strictly anaerobic conditions at 5°C. One of the strains grew under aerobic conditions (accordingly, a facultative anaerobe), whereas the remaining strains did not and, thus, were obligately anaerobic bacteria. The cells of most obligately anaerobic strains were Gram-positive motile rods. When obligately anaerobic strains were cultivated in 1/10 PYG broth at 5°C, acetate, butyrate, H_2_, and CO_2_ were typically detected as the end products of fermentation. Some strains additionally produced *n*-butanol as a major product. All of the obligately anaerobic strains grew at 10°C; however, the ability to grow at higher temperatures (20, 25, or 30°C) differed depending on the strains. Based on the upper limit of temperatures for growth, isolates were classified into the following four groups: group I, does not grow at higher than 20°C; group II, grows at 20°C with weak growth at 25°C; group III, grows at 25°C, but not at 30°C; group IV, grows at 30°C (Table 1).

### Phylogenetic ana­lysis of representative strains based on 16S rRNA gene sequences

**Fig. 1.**
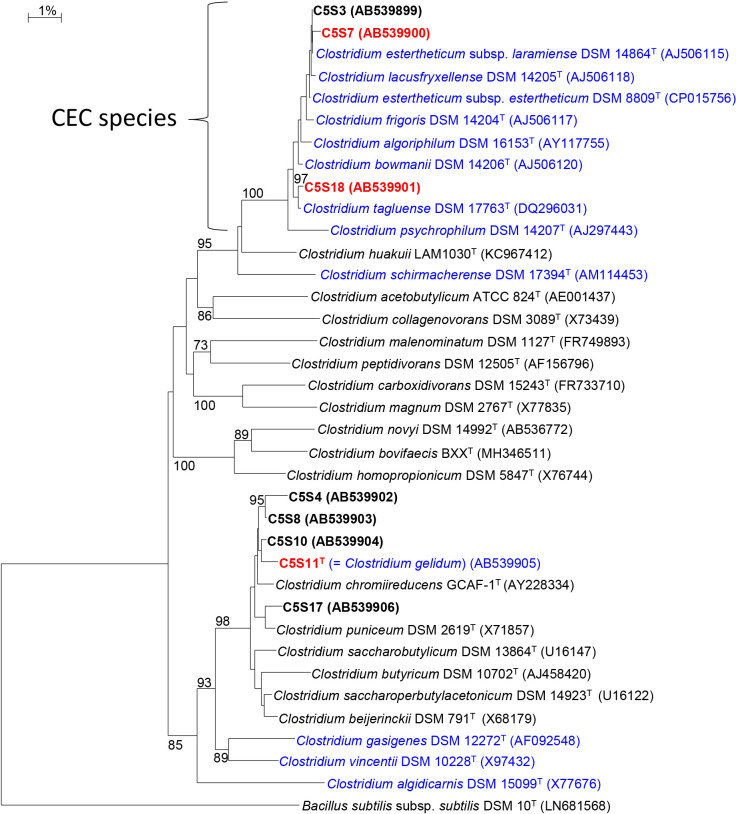
Neighbor-joining tree showing the phylogenetic relationship of strains isolated from enrichment cultures incubated at 5°C and related species in the genus *Clostridium* based on 16S rRNA gene sequences. Psychrotrophic species are highlighted in blue and other mesophilic species are in black. The strain numbers of isolates are shown in boldface and the three strains examined as representatives in the present study are highlighted in red. Bootstrap values (expressed as percentages of 1,000 replications) higher than 70% are shown at branch nodes. The sequence of *Bacillus subtilis* subsp. *subtilis* DSM 10^T^ (LN681568) was used as the outgroup. Bar, 1% estimated difference in the nucleotide sequence position.

Based on phenotypic properties and growth temperature ranges, eight obligately anaerobic strains (from group I: C5S3 and C5S7; group II, C5S18; group III, C5S4, C5S8, C5S10, and C5S11^T^; group IV, C5S17) were selected as representatives for the phylogenetic ana­lysis based on 16S rRNA gene sequences (Table 1). (Isolation sources: C5S3, C5S4, C5S7, and C5S8, the enrichment cultures inoculated with 10^–3^ diluted soil samples; C5S10 and C5S11^T^, 10^–4^ dilution; C5S17 and C5S18, 10^–5^ dilution). Sequence similarities with closely related species for the representative four strains are shown in Table S1. All closely related species of the strains belonged to the genus *Clostridium* and the phylogenies of the isolates mostly coincided with the grouping based on growth temperature ranges. Most of the known psychrotrophic species in the CEC group made an independent cluster in the genus *Clostridium* on the phylogenetic tree constructed and the two strains of group I (C5S3 and C5S7) were affiliated with the CEC group (Fig. 1). Strain C5S18 was also assigned to the same cluster on the phylogenetic tree. The sequence similarities of strain C5S7 with closely related species were 99.7% for *C. estertheticum* subsp. *laramiense* DSM 14864^T^ and 99.6% for *C. estertheticum* subsp. *estertheticum* DSM 8809^T^, both of which were psychrophilic species isolated from BPS samples. Other known psychrotrophic species were also related to strain C5S7 with high sequence similarities of approximately 99.0% (Table S1). Although strains C5S3, C5S7, and C5S18 were closely related to one another and formed a distinct group with known CEC species on the phylogenetic tree, the closest relative of strain C5S18 was *C. tagluense* A121^T^, a psychrotrophic species isolated from a permafrost sample (Suetin *et al.*, 2009), with a sequence similarity of 99.6% (Table S1). Sequence similarities between strain C5S18 and *C. estertheticum* subsp. *laramiense* DSM 14864^T^ and *C. estertheticum* subsp. *estertheticum* DSM 14864^T^ were 98.5 and 98.4%, respectively, while that between strains C5S7 and C5S18 was 98.5%, suggesting that the two strains belonged to different species according to the recommended prokaryotic species delineation value (98.7%) of 16S rRNA gene sequence similarities (Kim *et al.*, 2014; Chun *et al.*, 2018).

The closest relative of the four strains of group III was commonly *Clostridium chromiireducens* GCAF-1^T^ (Inglett *et al.*, 2011) with sequence similarities of 98.5–98.9% (Table S1). Although the four strains in the group had growth temperature ranges as psychrotrophs, they were affiliated with a cluster of mesophilic species in the genus *Clostridium* comprising species such as *C. chromiireducens*, *Clostridium puniceum* (Lund *et al.*, 1981), and *Clostridium beijerinckii* (Rainey *et al.*, 2009). The sequence similarity of strain C5S17 (group IV) with the closest relative *C. puniceum* was 98.9%. Although strain C5S17 was also assigned to the same cluster as the strains of group III, it grew at 30°C (Table 1), sharing the mesophilic property of phylogenetically related species. Based on these results, three strains (C5S7, C5S11^T^, and C5S18) were selected as representatives for further detailed physiological examinations of psychrotrophic anaerobic isolates.

### Growth temperature ranges and growth yields of three representative strains

**Fig. 2.**
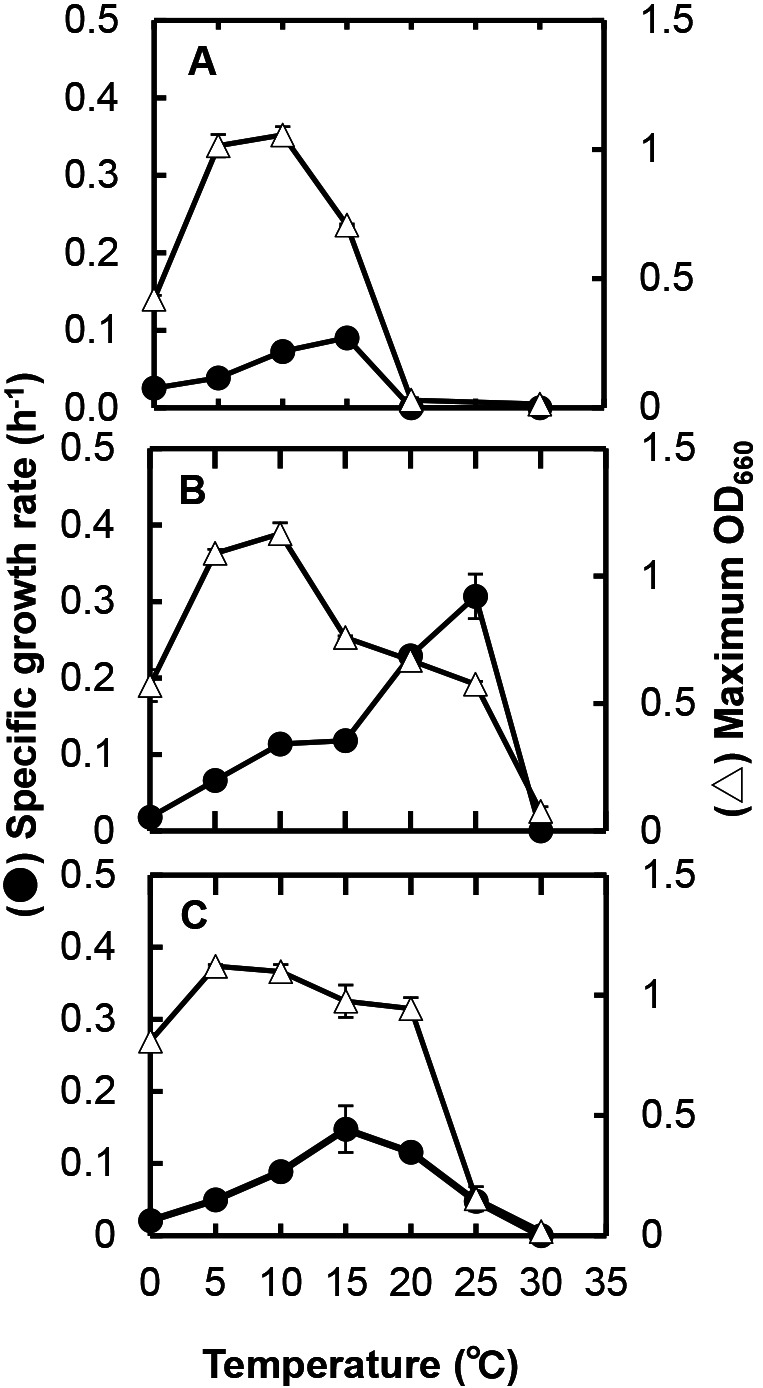
Specific growth rates and maximum OD_660_ values (growth yields) of strains C5S7 (A), C5S11^T^ (B), and C5S18 (C) at different temperatures. Each growth rate and the maximum OD_660_ value were assessed based on the growth curves cultivated in duplicate at each temperature. Error bars smaller than the sizes of the symbols are not visible.

The growth temperature ranges of the three strains were reexamined in more detail at intervals of 5°C (0, 5, 10, 15, 20, 25, and 30°C) and growth rates (μ) at the temperatures examined were assessed for each strain. The growth curves at different temperatures for strain C5S7 are shown in Fig. S1, and the growth rate and highest OD_660_ value (growth yield) at each temperature obtained are plotted in Fig. 2A. Although the growth rate was low (μ=0.025 h^–1^), strain C5S7 grew at 0°C and reached an OD_660_ of approximately 0.6 after 11–12‍ ‍d of cultivation. Strain C5S7 did not grow at 20°C, as shown in Table 1. The highest growth rate of strain C5S7 was obtained at 15°C (μ=0.090 h^–1^); however, growth stopped in the early stage of the incubation. Although the growth rate at 10°C (μ=0.073 h^–1^) was lower than that at 15°C, the growth yield reached the highest among all of the temperatures examined after cultivation for three to four days. The growth rate at 5°C (μ=0.039 h^–1^) was approximately half that at 10°C, whereas growth yields were similar. Based on these results, the optimum temperature for the stable growth or proliferation of strain C5S7 was considered to be approximately 5–10°C.

The growth rate and highest OD_660_ value at each temperature for strains C5S11^T^ and C5S18 obtained from the same growth experiments are shown in Fig. 2B and 2C, respectively. Regarding strain C5S11^T^, as already reported by Honma *et al.* (2022), the growth temperature range of the strain was between 0 (μ=0.018 h^–1^) and 25°C with the highest growth rate at 25°C (μ=0.307 h^–1^); however, growth at a temperature higher than 15°C stopped in the early stage of the incubation and OD_660_ values declined soon thereafter. The growth rate at 10°C was 0.114 h^–1^ and the final growth yield became the highest at the temperatures examined, indicating that the optimum temperature of the strain was approximately 5–10°C. The growth temperature range of strain C5S18 ranged between 0 (μ=0.021 h^–1^) and 25°C (μ=0.048 h^–1^). Although the highest growth rate was noted at 15°C (μ=0.148 h^–1^), similar high growth yields were obtained between 5 and 20°C. The growth of the strain at 25°C was poor, and it was not possible to transfer the cells grown at 25°C to fresh medium for cultivation at the same temperature. Therefore, the optimum temperature of strain C5S18 was considered to be approximately 15°C.

**Fig. 3.**
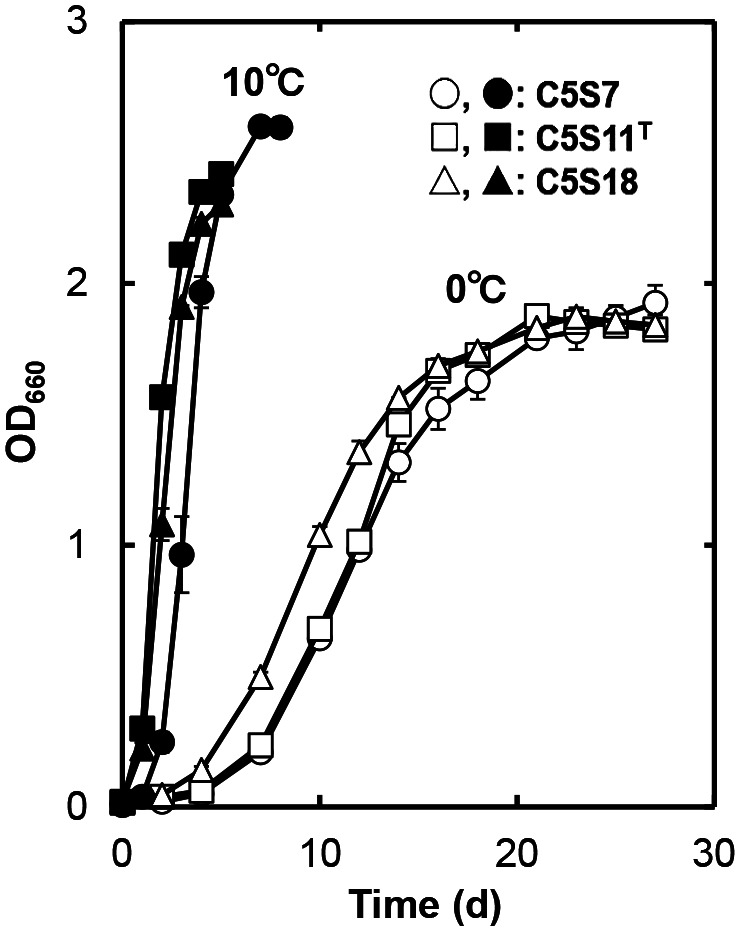
Growth curves of strains C5S7, C5S11^T^, and C5S18 at 0 and 10°C cultivated in PYG broth. Average OD_660_ values from duplicate cultivations are shown. Error bars smaller than the sizes of the symbols are not visible.

When the three strains were cultivated at 0 and 10°C in PYG broth as a nutrient-rich medium (Fig. 3), they also grew with similar growth rates to those in 1/10 PYG broth. The growth yield of each strain was >2.5-fold higher than that in 1/10 PYG at the respective temperature. Based on these results, PY broth was used as the basal medium for the‍ ‍physiological characterization of the three strains unless otherwise indicated.

### Diversity of substrate utilization and end products of three representative strains as fermentative anaerobic bacteria

Further phenotypic examinations were conducted on strains C5S7, C5S11^T^, and C5S18 to investigate their functions in cold soil and confirm their identification. Although the phenotypic characteristics of strain C5S11^T^ were comprehensively described by Honma *et al.* (2022), some key properties of the strain were shown below for comparisons with other isolates. The cells of the three strains were obligately anaerobic Gram-positive spore-forming rods. Strains C5S7 and C5S18 showed similar cellular characteristics (Fig. S2A and C). The cells of strain C5S11^T^ were straight rods and produced spores after a long cultivation on agar slant cultures (Fig. S2B). The growth of strain C5S7 in PY broth was weak, whereas it actively grew in PYG, indicating saccharolytic metabolism by the strain. Strain C5S7 utilized various carbohydrates and produced butyrate, *n*-butanol, and abundant gases (H_2_ and CO_2_) as major products with small amounts of acetate from the sugars utilized (Table 2). A trace amount of ethanol was also produced from most of the carbohydrates utilized. Furthermore, strain C5S11^T^ was saccharolytic and decomposed various carbohydrates, including polysaccharides, such as inulin, pectin, starch, and xylan, as easily fermentable substrates. The strain produced acetate and butyrate as well as abundant gases (H_2_ and CO_2_) as fermentation products from the carbohydrates utilized (Honma *et al.*, 2022). In contrast to the two strains, strain C5S18 grew well in PY broth without added carbohydrates and produced acetate with low amounts of butyrate, H_2_, and CO_2_. Substrate utilization, including carbohydrates, organic acids, and amino acids, by the strain was comprehensively examined to confirm the characteristics of the strain (Table 2 and S2). Although the addition of glucose and maltose to PY broth slightly increased butyrate production by the strain, the addition of other carbohydrates did not stimulate the growth and production of the acid more than PY broth, indicating that the strain was essentially asaccharolytic and utilized proteinaceous substrates, such as casein hydrolysates (trypticase), in PY broth. Strain C5S18 produced acetate from an amino acid, serine, out of the 20 amino acids examined. Organic acids (fumarate, malate, and pyruvate) also enhanced acetate production by the strain (Table S2).

### Identification of three representative strains based on phylogeny and phenotypic properties

The major characteristics of strains C5S7, C5S11^T^, and C5S18 and their closest relatives are shown in Table 3. Similarities in the 16S gene sequences of strains C5S7 and C5S18 with their closest relatives were 99.7 and 99.6% (Table S1), respectively, and higher than the threshold values recommended for bacterial species delineation. The cellular characteristics and physiological properties of strain C5S7 were consistent with those of *C. estertheticum* (Table 3). Although strain C5S18 was phylogenetically assigned to the same large cluster as that of strain C5S7 (Fig. 1), the closest relative was a different species, *C. tagluense*. The asaccharolytic property of strain C5S18 corresponded to that of *C. tagluense* (Suetin *et al.*, 2009). Based on their physiological properties in addition to their growth temperature ranges and phylogenies, strains C5S7and C5S18 were identified as the psychrotrophic species, *C. estertheticum* and *C. tagluense*, respectively. The basic physiological properties of strains C5S7 and C5S18 markedly differed in spite of their closely related phylogenies, indicating the different functional roles of the bacterial groups represented by these strains.

Based on the polyphasic data obtained for strain C5S11^T^, *Clostridium gelidum* sp. nov. was already validly described with strain C5S11^T^ as the type strain (Honma *et al.*, 2022).

## Discussion

Previous studies on psychrotrophic anaerobes were exclusively conducted for ecosystems in extremely cold areas, such as polar regions and permafrost soil, or in vacuum-packed chill-stored meat. In the present study, obligately anaerobic psychrotrophic strains of different species in the genus *Clostridium* were isolated from rice field soil in Japan managed under a general cultivation method for a long time. The results obtained indicated that various cold-adapted anaerobic bacteria affiliated with different phylogenetic groups in the genus *Clostridium* inhabited the soil.

Strain C5S7 was saccharolytic, utilized various mono- and disaccharides, and produced fatty acids and *n*-butanol as well as abundant gases. Strain C5S11^T^ was also saccharolytic and utilized diverse carbohydrates, including different types of plant polysaccharides. Strain C5S18 was asaccharolytic and grew actively in PY broth without the addition of carbohydrates. Since strain C5S17 grew at 30°C (Table 1) and possessed similar properties to the closest mesophilic relative *C. puniceum* (98.9% sequence similarity, Table S1), we did not examine the characteristics of the strain in detail. The cells of strain C5S17 were obligately anaerobic, Gram-positive, spore-forming, motile rods. The strain grew at 5–10°C with similar growth rates as those of strain C5S11^T^. It was saccharolytic and produced acetate, butyrate, H_2_, and CO_2_ from glucose, suggesting that the bacterial group represented by strain C5S17 is involved in the decomposition of carbohydrates under cold conditions in soil. Therefore, the obligately anaerobic, cold-adapted clostridial strains isolated from soil had different growth temperature ranges, substrate utilization, or compositions of fermentation products.

*C. estertheticum* is a psychrophilic anaerobe that causes BPS in vacuum-packed meat. *C. estertheticum* has often been isolated from or detected in meat or spoiled meat samples (Cavill *et al.*, 2011; Bonke *et al.*, 2016; Wambui and Stephan, 2019; Zhang *et al.*, 2019; Wambui *et al.*, 2021, 2022); however, the original environmental habitats or contamination routes of the bacterium to meat remain unclear (Esteves *et al.*, 2021). Although the closest relative of strain C5S18 was *C. tagluense*, which is phylogenetically included in the CEC group, the physiological characteristics of strain C5S18 or *C. tagluense* markedly differed from those of strain C5S7 or *C. estertheticum* (Suetin *et al.*, 2009). *C. tagluense* or *C. tagluense*-like strains were recently detected in or isolated from various meat samples (Dorn-In *et al.*, 2018, 2022; Wambui *et al.*, 2022). Lower gas production by these *C. tagluense*-like isolates than by *C. estertheticum* strains (Wambui *et al.*, 2022) coincided with the results obtained for strains C5S7 and C5S18 in the present study (Table 2). The draft genome sequences of strains related to *C. tagluense* or *C. tagluense*-like species showed that these organisms encode 50% of the carbohydrate-active enzymes of *C. estertheticum* subsp. *laramiense* DSM 14864^T^ (Murakami *et al.*, 2019; Palevich *et al.*, 2020). *C. tagluense* strains have a smaller number of glycoside hydrolase (GHs) genes in their genomes than *C. estertheticum* strains (Wambui *et al.*, 2022). Since *C. tagluense* was originally isolated from a permafrost sample in the High Arctic of Canada, the characteristics of strain C5S18 were comprehensively examined in the present study to confirm the‍ ‍de­scription of the strain as *C. tagluense* isolated from Japanese rice field soil (Table S2). The isolation of a strain of *C. tagluense* from rice field soil located in the temperate region at a mid-latitude in Japan is consistent with the isolation of *C. tagluense* strains from various meat or chill-stored meat samples (Dorn-In *et al.*, 2022; Wambui *et al.*, 2022) and suggested the wide distribution of the species in anoxic environments on the earth. The upper limit of temperature for the growth of *C. tagluense* was higher than that of *C. estertheticum* (Table S1) and corresponded to differences in the growth temperature ranges of strains C5S7 and C5S18. The higher limit of temperature for growth needs to be advantageous for *C. tagluense* to prevail in various environments. The ultimate origin of the psychrotrophic anaerobic species, *C. tagluense*, in the temperate region remains unclear.

*C. estertheticum* and *C. tagluense* are closely related to each other based on their 16S rRNA gene sequences and both are assigned to a single clade in the genus *Clostridium* as the CEC group. Furthermore, the cell morphologies of both species are similar, as shown for strains C5S7 and C5S18 (Fig. S2). These properties of the two species increase the difficulties associated with distinguishing isolates of the two species. Genome data obtained from various CEC species or isolates showed distinct differences in the distribution of genes between the two species (Wambui *et al.*, 2022). However, physiological differences in the two species suggest that it is practically useful to examine their growth patterns in medium with or without glucose in order to distinguish *C. tagluense* strains from *C. estertheticum* strains as a routine examination for CEC isolates.

The psychrotrophic anaerobic bacteria causing BPS grow at temperatures lower than 15–20°C. Therefore, these bacteria may proliferate in most seasons as indigenous anaerobic bacteria in the presence of growth substrates under anoxic environments, including arable lands. Psychrotrophic clostridial species generally produce spores, which enables the survival of the species under unfavorable conditions. The spores of these species produced in anoxic environments may be dispersed as soil dust and are eventually transferred to the processing steps of vacuum-packed meat, where they induce spoilage as contaminants. The strains examined in the present study were isolated from enrichment cultures inoculated with 10^–3^–10^–5^ diluted soil samples and incubated at 5°C. Therefore, a trace amount of the soil sample had sufficient potential to cause the spoilage of meat under anaerobic and chilled conditions. *C. estertheticum* has been isolated from bovine fecal samples as well as other farm environmental samples (Esteves *et al.*, 2021; Wambui *et al.*, 2021). Since psychrotrophic anaerobic species do not grow at temperatures higher than 30°C, they are not able to grow in the gastrointestinal tracts of domestic mammals, the body temperature of which is 38–39°C. Therefore, even if the spores of these bacteria, which were ingested from some environmental sources, enter the digestive tracts of animals and are released outside the bodies as feces, mammalian bodies or digestive tracts are not likely to be the major habitat for the proliferation of psychrotrophic anaerobic bacteria causing BPS.

Cold stress or a rapid temperature drop (cold shock) induces cold-induced proteins (Cips), such as cold shock proteins (RNA chaperone, Csps), in many bacterial species (Keto-Timonen *et al.*, 2016; Zhang and Gross, 2021). Although strain C5S11^T^ was regarded as a psychrotrophic bacterium based on the growth temperature range, the phylogenetically related species were mesophilic species. Honma *et al.* (2022) reported that according to the genome annotation data of strain C5S11^T^, two genes encoding CspA (WP_224037979.1) and cold shock domain-containing protein or Csp-like protein (CspLA) (WP_224037872.1) were present in the genome. Homologues were detected in the genomes of true psychrophilic CEC members, such as *C. estertheticum*, *C. frigoris*, and *C. bowmanii*, for both genes in the NCBI database using the Protein BLAST search. In contrast, homologues of these genes were not identified in phylogenetically related mesophilic species with some exceptional species. These genome data suggest that the presence of Cips represented by these Csps provides the psychrotrophic physiology of strain C5S11^T^ or *C. gelidum*.

The present results showed that a bacterial community consisting of these diverse, cold-adapted anaerobic, fermentative bacteria has the ability to degrade various organic compounds (namely, carbohydrates, including polysaccharides, proteinaceous compounds, such as amino acids, and organic acids) in rice field soil at soil temperatures lower than approximately 5°C during the cold season. The products of these fermentative bacteria may serve as growth substrates or electron donors for other anaerobic microbes, including Fe(III)- or sulfate-reducing bacteria, and methanogens in soil. Further investigations on the distribution of psychrotrophic anaerobic bacteria are warranted to clarify their microbial community compositions and ecological functions under cold conditions in rice field soil in temperate regions.

## Citation

Honma, S., Ueki, A., Ichimura, A., Suzuki, K., Kaku, N., and Ueki, K. (2023) Phylogeny and Physiological Diversity of Cold-adapted Anaerobic Bacteria Isolated from Rice Field Soil in Japan. *Microbes Environ ***38**: ME22109.

https://doi.org/10.1264/jsme2.ME22109

## Supplementary Material

Supplementary Material

## Figures and Tables

**Table 1.** Differences in growth temperature ranges of isolates.

Group	Temperature (°C)					Representative strains
	5	10	20	25	30	
I	+	+	–	–	–	C5S3, 7
II	+	+	+	w+	–	C5S18
III	+	+	+	+	–	C5S4, 8, 10, 11
IV	+	+	+	nd	+	C5S17

Growth: +, positive; w+, weakly positive or slow growth; –, negative; nd, not determined.

**Table 2.** Major fermentation end products from various substrates of strains C5S7, C5S11^T^, and C5S18.

Strain	Basal medium	Substrate	Fermentation products (mmol L^–1^)				
			Acetate	Butyrate	*n*-Butanol	H_2_	CO_2_
C5S7	PY	None	—	2.7	tr	2.3	2.9
		Arabinose	2.4	14.5	5.2	10.1	24.3
		Xylose	tr	12.1	3.8	10.0	19.5
		Glucose	3.3	9.9	18.5	10.1	28.4
		Cellobiose	3.1	17.2	14.9	15.0	36.3
		Maltose	2.6	13.3	5.7	13.5	22.5
		Starch	4.4	17.2	10.4	15.8	30.4
C5S11^T^*	PY	None	—	2.0	—	7.2	4.3
		Arabinose	7.5	18.5	—	32.4	15.2
		Xylose	7.8	18.9	—	30.9	16.4
		Glucose	8.8	19.3	—	23.7	14.0
		Cellobiose	10.8	17.2	—	33.4	16.1
		Maltose	10.8	18.0	—	35.8	18.6
		Inulin	11.2	19.9	—	41.1	22.3
		Pectin	7.3	11.0	—	29.2	14.1
		Starch	11.2	18.6	—	33.7	18.5
		Xylan	9.3	10.9	—	26.3	15.1
C5S18	PY	None	18.8	1.8	—	2.7	6.7
		Glucose	10.8	4.9	—	17.9	12.0
		Maltose	12.0	5.2	—	6.0	12.9
		Fumarate	26.3	2.1	—	5.4	9.6
		Malate	24.6	2.1	—	6.7	12.3
	1/10 PY	None	3.3	—	—	1.9	3.5
		Serine	17.5	—	—	6.3	10.8

Values are the averages of duplicate cultivations at 10°C. Cultivation periods: strains C5S11^T^ and C5S18, 5–6 days; strain C5S7, approximately 10 days. —, Not detected; tr, trace. Strain C5S7 did not decompose polysaccharides, except for starch, while strain C5S18 did not decompose any of the polysaccharides examined (inulin, pectin, starch, and xylan). *, This study and Honma *et al.* (2022).

**Table 3.** Comparison of major characteristics of strains C5S7, C5S11^T^, and C5S18 with those of their respective closest relatives.

Characteristics	C5S7	Clostridium estertheticum*	C5S11^T^	*Clostridium chromiireducens*	C5S18	*Clostridium tagluense*
Original isolation source	Rice field soil	Chilled vacuum-packed meat	Rice field soil	Chromium-contaminated soil	Rice field soil	Ice-bonded sand from permafrost
Cellular characteristics						
Morphology	Rods	Rods	Short rods	Short to long rods	Rods	Rods
Gram staining	+	+	+	+	+	+
Size (μm)						
Width	0.9–1.2	1.3–1.5	0.7–0.9	1.0–1.5	0.8–1.0	1.0–1.2
Length	3.1–5.2	2.4–6.0	2.1–4.0	4.0–7.0	1.4–4.0	3.0–10.0
Spore	+ (T)	+ (ST to T)	+ (ST to T)	+ (T to ST)	+ (T)	+ (ST)
Motility	+	+	+	+	+	+
Catalase	–	–	–	nr	–	–
Temperature range for growth (°C)	0–15	21 (upper limit)	0–25	10–40	0–25	4–28
Optimum growth (°C)	10	15	10	35–40	15	15–20
Substrate utilization						
Arabinose	+	+	+	+	–	–
Xylose	+	+	+	+	–	–
Glucose	+	+	+	+	w+	+
Cellobiose	+	+	+	+	–	–
Maltose	+	+	+	+	w+	+
Inulin	–	nr	+	+	–	nr
Pectin	–	nr	+	+	–	nr
Starch	+	+	+	nr	–	+
Xylan	–	nr	+	+	–	nr
Fumarate	nd	nr	nd	nr	w+	+
Malate	nd	nr	nd	nr	w+	+
Peptone (trypticase)	–	nr	–	–	+	+
Fermentation end products	A, B, 2, 4, H_2_, CO_2_	A, B, f, l, 2, 4, H_2_, CO_2_	A, B, H_2_, CO_2_	A, B, H_2_, CO_2_	A, B, H_2_, CO_2_	A, B, f, v, 2, H_2_, CO_2_
References	This study	Collins *et al.*, 1992; Spring *et al.*, 2003	Honma *et al.*, 2022	Inglett *et al.*, 2011	This study	Suetin *et al.*, 2009

+, positive; w+, weakly positive; –, negative; T, terminal; ST, subterminal; nd, not determined; nr, not reported. Products: f, formate; A, acetate; B, butyrate; v, valerate; l, lactate; 2, ethanol; 4, butanol. Lowercase letters indicate minor products. *, Common characteristics of the two subspecies are shown.
