# Supplementary material for: Phylogeny and Physiological Diversity of Cold-adapted Anaerobic Bacteria Isolated from Rice Field Soil in Japan
Source: Microbes Environ. 2023 May 11;38(2):ME22109. doi: 10.1264/jsme2.ME22109 (PMC10308237; doi:10.1264/jsme2.ME22109)
Supplement: Supplementary file 1 — Supplementary Material [file 38_22109_s1.pdf]

Microbes and Environments

Supplementary materials (2 figures and 2 tables)

Phylogeny and Physiological Diversity of Cold-Adapted Anaerobic Bacteria Isolated from Rice Field Soil in Japan

Sachi Honma, Atsuko Ueki*, Akio Ichimura, Kouki Suzuki, Nobuo Kaku, and Katsuji Ueki

Faculty of Agriculture, Yamagata University, Tsuruoka, 997-8555, Japan

Running headline: Psychrotrophic *Clostridium* from paddy soil

*Author for correspondence: Atsuko Ueki. Tel: +81 235 28 2846

E-mail: uatsuko@tds1.tr.yamagata-u.ac.jp

Table S1. Closely related species of the representative isolates based on the 16S rRNA gene sequence similarities, and their growth temperature ranges and original isolation sources.

Isolate	Closely related species	Accession No.	Sequence similarity (%)	Temperature for growth (°C)		Original isolation source
				Range	Optimum	
C5S7	*Clostridium estertheticum* subsp. *laramiense* DSM 14864^{T}	AJ506115	99.7	-3–21	15	Vacuum-packed meat
	Clostridium estertheticum subsp. *estertheticum* DSM 8809^{T}	CP015756	99.6	13 (upper limit)	6–8	Vacuum-packed meat
	Clostridium lacusfryxellense DSM 14205^{T}	AJ506118	99.5	15 (upper limit)	8–12	Antarctic microbial mat
	Clostridium frigoris DSM 14204^{T}	AJ506117	99.2	11 (upper limit)	5–7	Antarctic microbial mat
	Clostridium algoriphilum 14D1^{T}	AY117755	99.0	-5–20	5	Permafrost of the Tundra region
	Clostridium bowmanii DSM 14206^{T}	AJ506120	98.8	20 (upper limit)	12–16	Antarctic microbial mat
	Clostridium tagluense A121^{T}	DQ296031	98.6	4–28	15–20	Permafrost in the Canadian High Arctic
C5S11^{T}	*Clostridium chromiireducens* GCAF-1^{T}	AY228334	98.5	10–40	35–40	Chromium-contaminated soil
	Clostridium puniceum DSM 2619^{T}	X71857	98.3	7–39	30	Rotting potatoes
	Clostridium saccharobutylicum NCP 262^{T}	U16147	98.1	15–40	35	Industrial fermentation plant
	Clostridium saccharoperbutylacetonicum N1-4(HMT)^{T}	U16122	98.1	nr	25–35	Solvent-producing industrial setting
	Clostridium beijerinckii DSM 791^{T}	X68179	98.0	25–45	37	Soil
C5S17	*Clostridium puniceum* DSM 2619^{T}	X71857	98.9	7–39	30	Rotting potatoes
	Clostridium saccharoperbutylacetonicum N1-4(HMT)^{T}	U16122	98.3	nr	25–35	Solvent-producing industrial setting
	Clostridium chromiireducens GCAF-1^{T}	AY228334	98.1	10–40	35–40	Chromium-contaminated soil
	Clostridium beijerinckii DSM 791^{T}	X68179	98.0	25–45	37	Soil
C5S18	*Clostridium tagluense* A121^{T}	DQ296031	99.6	4–28	15–20	Permafrost in the Canadian High Arctic
	Clostridium algoriphilum 14D1^{T}	AY117755	99.0	-5–20	5	Permafrost of the Tundra region
	Clostridium bowmanii DSM 14206^{T}	AJ506120	98.9	20 (upper limit)	12–16	Antarctic microbial mat
	Clostridium estertheticum subsp. *laramiense* DSM 14864^{T}	AJ506115	98.5	-3–21	15	Vacuum-packed meat
	Clostridium estertheticum subsp. *estertheticum* DSM 8809^{T}	CP015756	98.4	13 (upper limit)	6–8	Vacuum-packed meat

nr, Not reported.

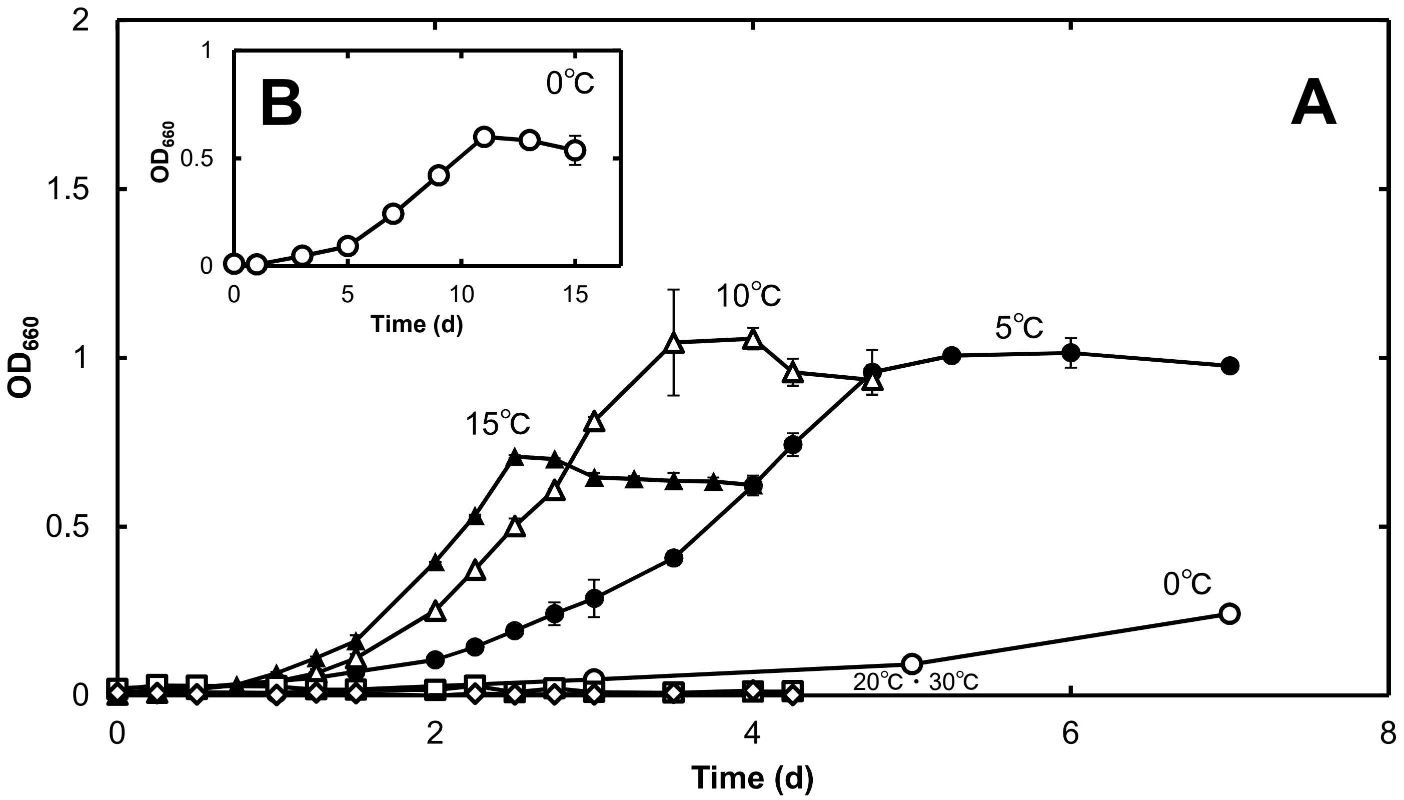

Fig. S1. Growth of strain C5S7 in 1/10 PYG broth at different temperatures.

(A) at different temperatures and (B) at 0℃ for the prolonged cultivation period. Average OD_{660} values from the cultivations in duplicate are shown. Error bars smaller than the sizes of the symbols are not visible.

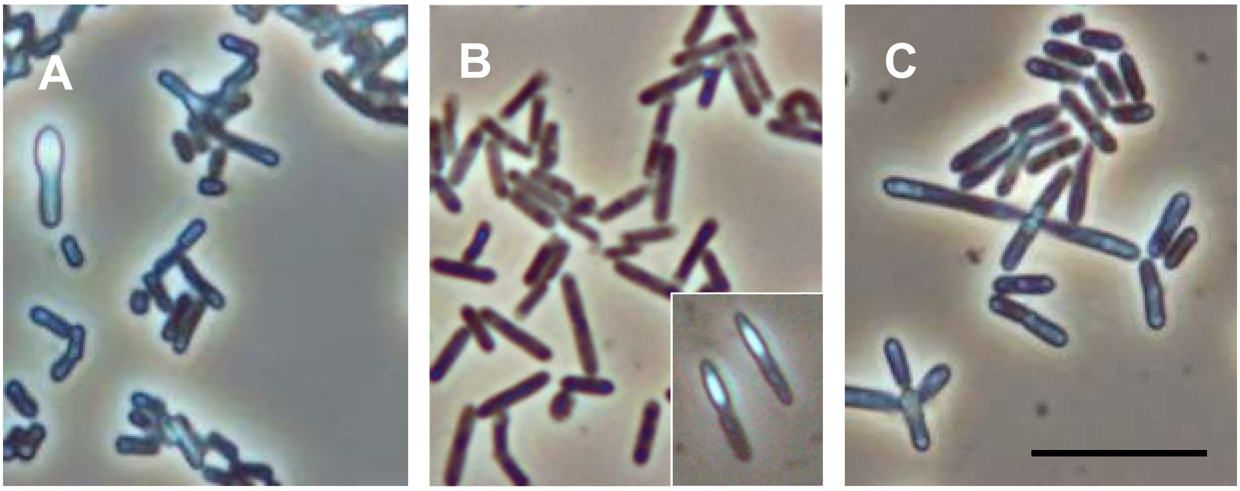

Fig. S2. Phase-contrast photomicrographs of cells of strains C5S7 (A), C5S11^{T} (B), and C5S18 (C). The insert of (B) shows spores of strain C5S11^{T}. Bar, 10 μm.

Table S2. Characteristics of strain C5S18 comprehensively examined in the present study.

Cellular characteristics	Gram-stain-positive, spore-forming, motile rods
Temperature range for growth (optimum) (°C)	0–25 (15)
pH range for growth (optimum)	6.2–8.1 (6.9)
NaCl range for growth (optimum) (%, w/v)	0–2 (0)
Fermentation products	Acetate, butyrate, isovalerate, H_2, CO_2
Catalase	-
Oxidase	-
Hydrolysis of esculin	-
Hydrolysis of gelatin	-
Production of H_2S	+
Production of indole	-
Nitrate reduction	-
Iron reduction	-
Major cellular fatty acids (%)	$C_{16:1}$ω7cDMA (23.0), $C_{16:0}$ (19.7), $C_{16:1}$ω7c (12.0), $C_{14:0}$ (10.6), C16:1ω9c (4.7)
Diagnostic diamino acid in the peptidoglycan	*meso*-DAP
Genome DNA G+C content (mol%) (HPLC)	32.2

Substrates utilized: Trypticase, serine

Substrates weakly utilized: glucose, maltose, pyruvate, fumarate, malate

Substrates examined but not-utilized:

[Carbohydrates] arabinose, ribose, xylose, fructose, galactose, mannose, rhamnose, cellobiose, lactose, melibiose, saccharose, sorbose, trehalose, melezitose, raffinose, CMC, dextrin, glycogen, inulin, pectin, starch, xylan, amygdalin, esculin, salicin, dulcitol, glycerol, inositol, mannitol, sorbitol

[Amino acids] alanine, arginine, asparagine, aspartate, glutamate, glutamine, glycine, histidine, isoleucine, leucine, lysine, methionine, ornithine, phenylalanine, proline, threonine, tryptophan, tyrosine, valine

[Organic acids] lactate, succinate
